# Supplementary material for: SPRINT: ultrafast protein-protein interaction prediction of the entire human interactome
Source: BMC Bioinformatics. 2017 Nov 15;18:485. doi: 10.1186/s12859-017-1871-x (PMC5688644; doi:10.1186/s12859-017-1871-x)
Supplement: Supplementary file 2 — This file contains the ROC and PR curves for all tests. (PDF 4782 KB) [file 12859_2017_1871_MOESM2_ESM.pdf]

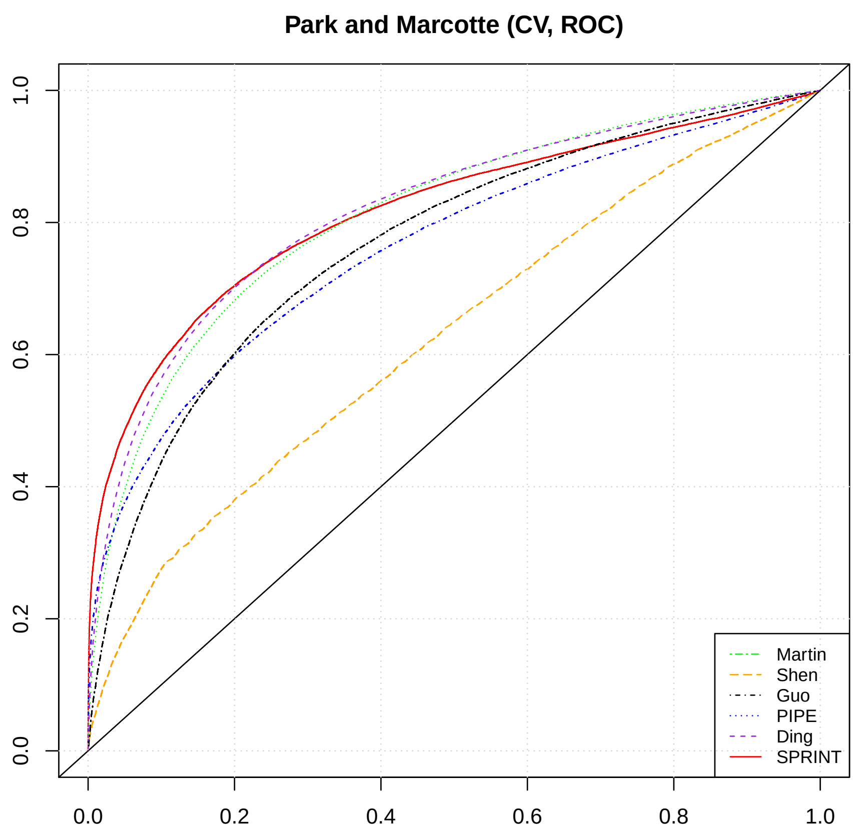
Park and Marcotte (CV, ROC)
0.0
0.2
0.4
0.6
0.8
1.0
Martin
Shen
Guo
PIPE
Ding
SPRINT

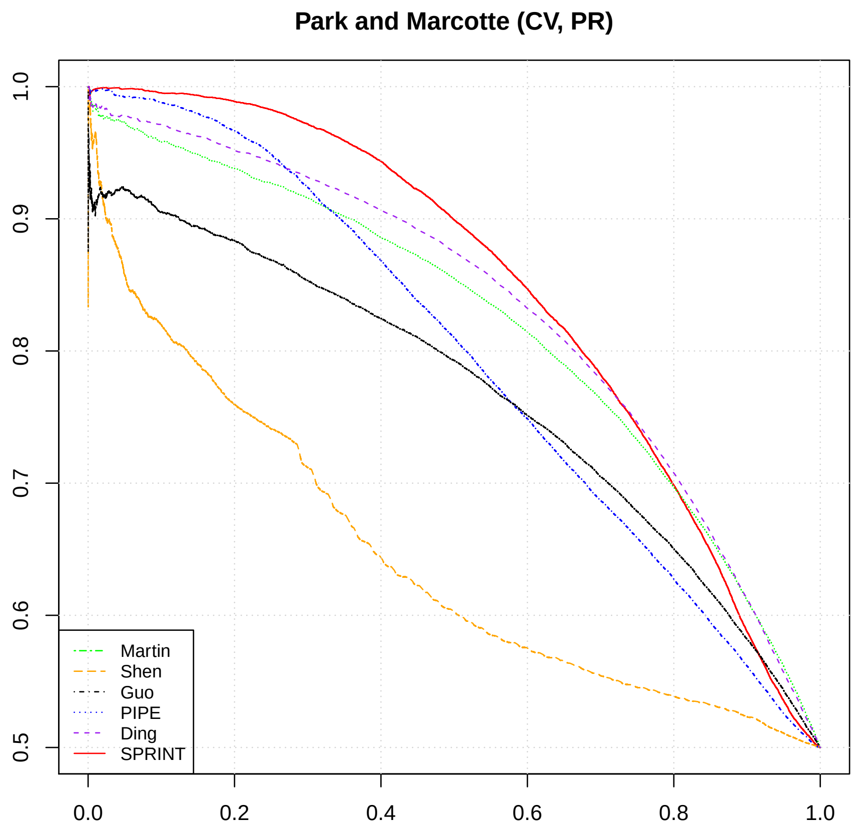
Park and Marcotte (CV, PR)
0.5
0.6
0.7
0.8
0.9
1.0
0.0
0.2
0.4
0.6
0.8
Martin
Shen
Guo
PIPE
Ding
SPRINT

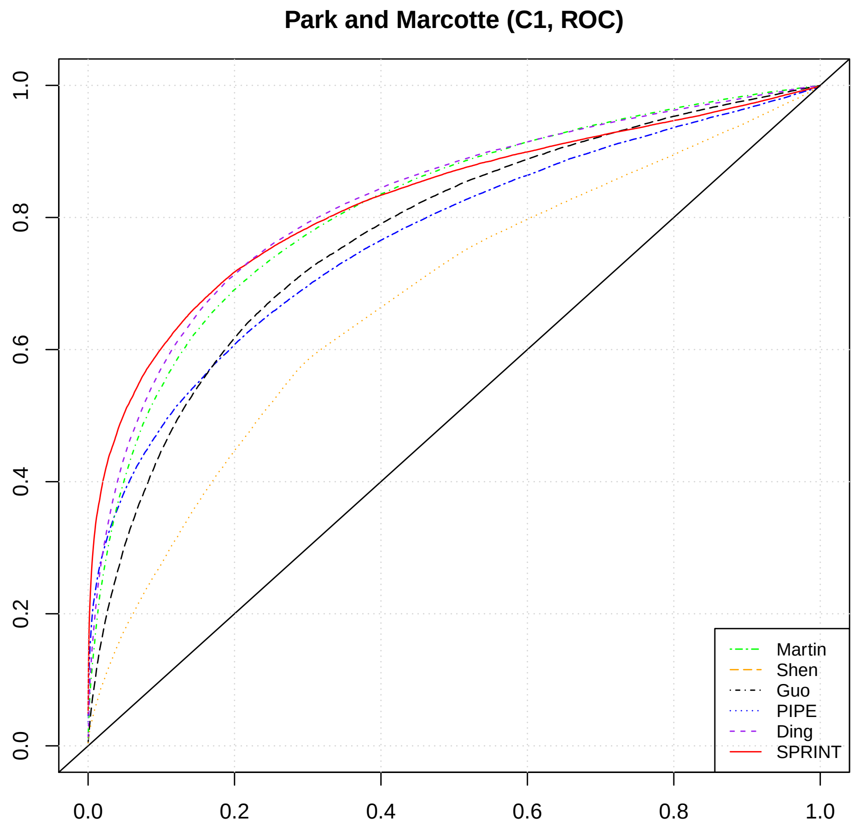
Park and Marcotte (C1, ROC)
0.0
0.2
0.4
0.6
0.8
1.0
Martin
Shen
Guo
PIPE
Ding
SPRINT

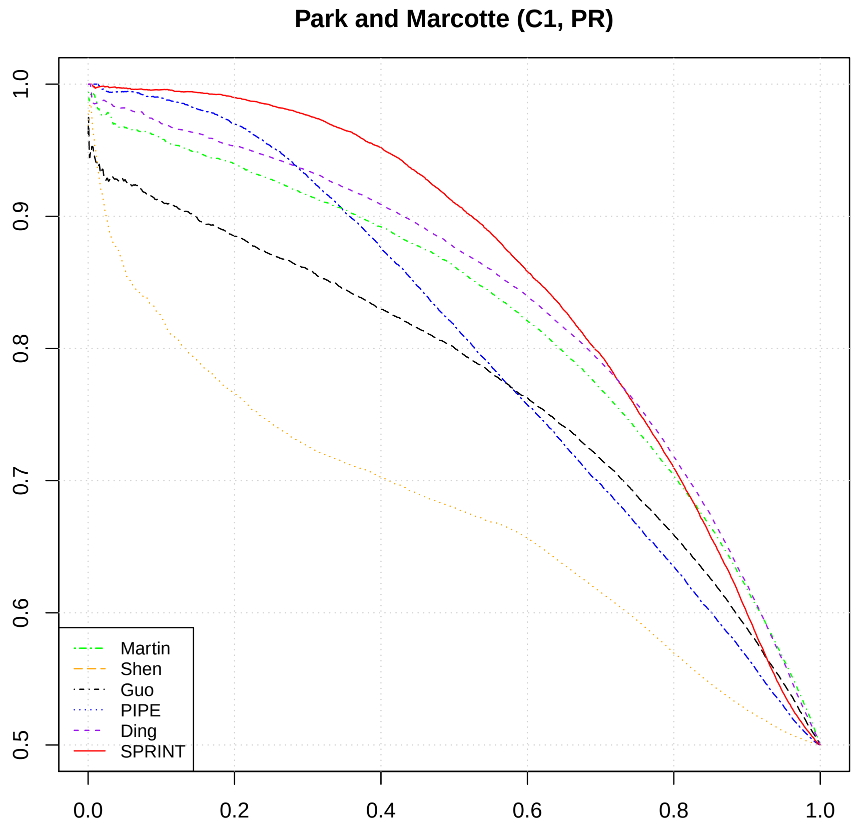
Park and Marcotte (C1, PR)
0.5
0.6
0.7
0.8
0.9
1.0
0.0
0.2
0.4
0.6
0.8
Martin
Shen
Guo
PIPE
Ding
SPRINT

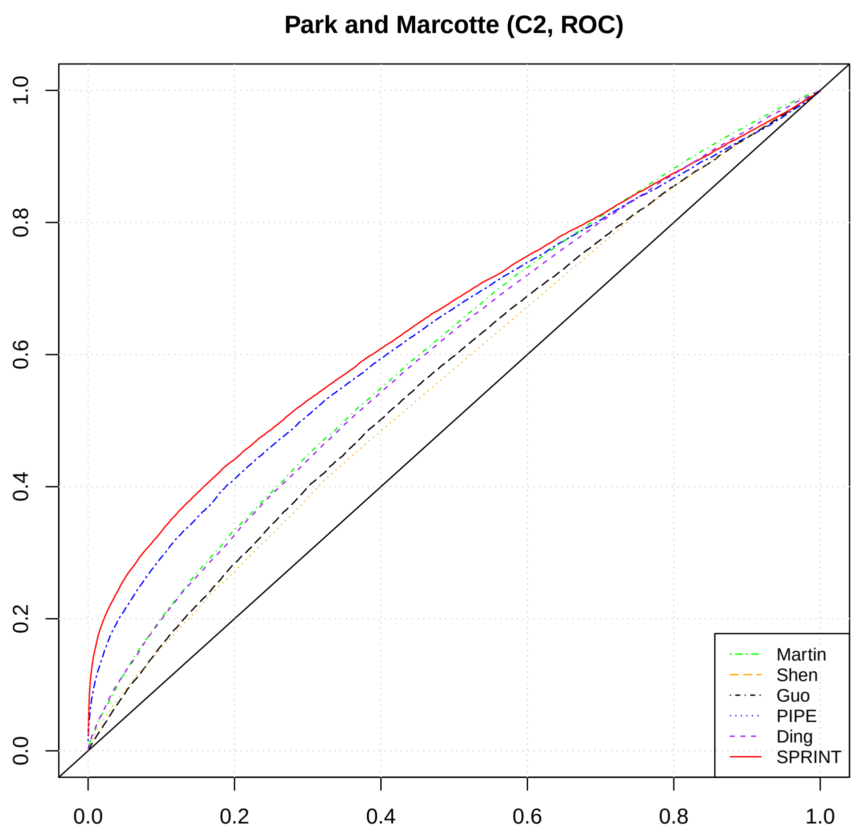
Park and Marcotte (C2, ROC)
0.0
0.2
0.4
0.6
0.8
1.0
Martin
Shen
Guo
PIPE
Ding
SPRINT

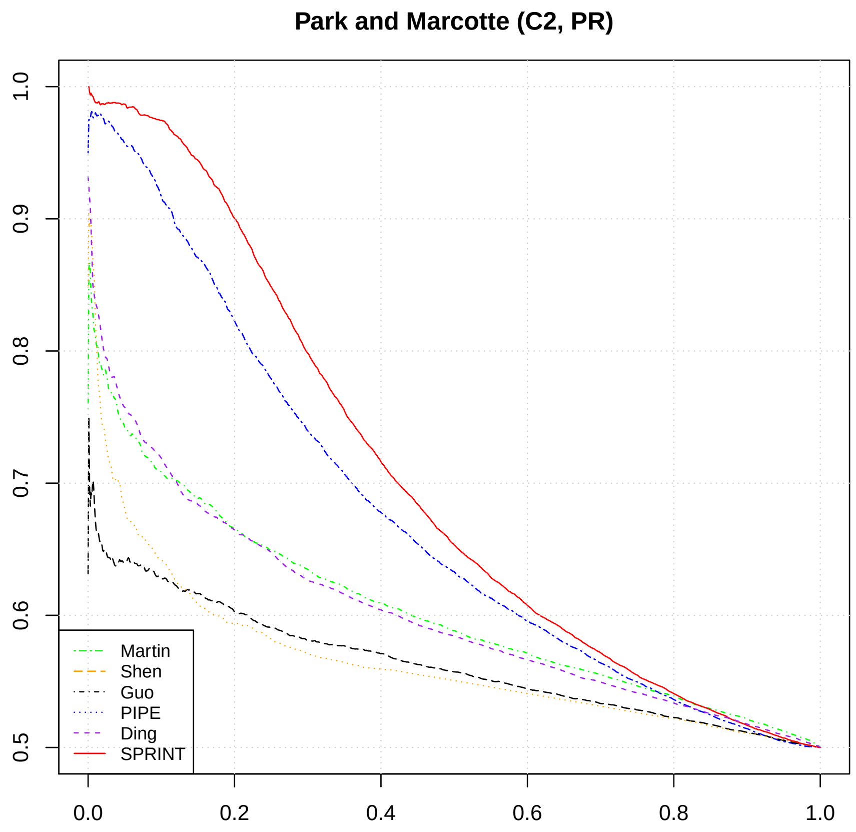
Park and Marcotte (C2, PR)
0.5
0.6
0.7
0.8
0.9
1.0
0.0
0.2
0.4
0.6
0.8
1.0
Martin
Shen
Guo
PIPE
Ding
SPRINT

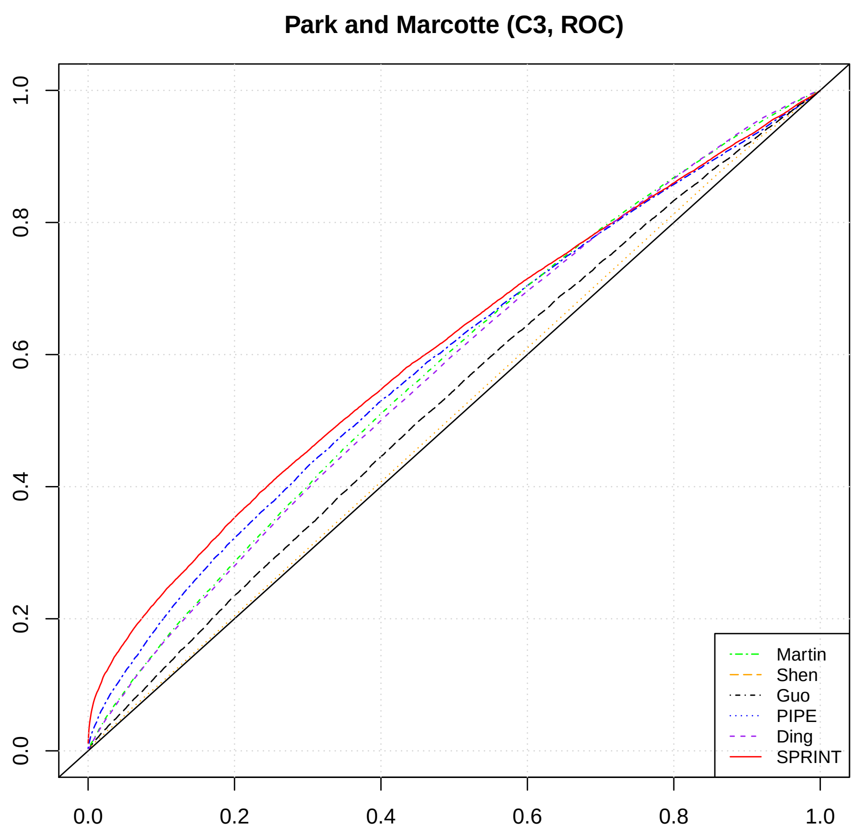
Park and Marcotte (C3, ROC)
Martin
Shen
Guo
PIPE
Ding
SPRINT
0.0
0.2
0.4
0.6
0.8
1.0

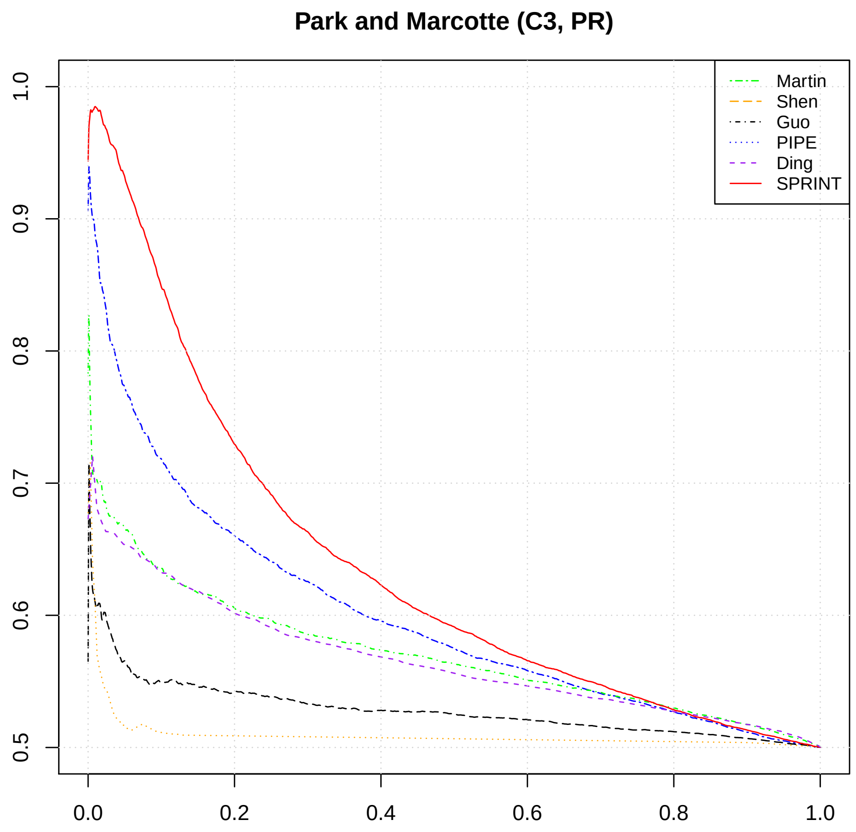
Park and Marcotte (C3, PR)
Martin
Shen
Guo
PIPE
Ding
SPRINT
0.5
0.6
0.7
0.8
0.9
1.0
0.0
0.2
0.4
0.6
0.8

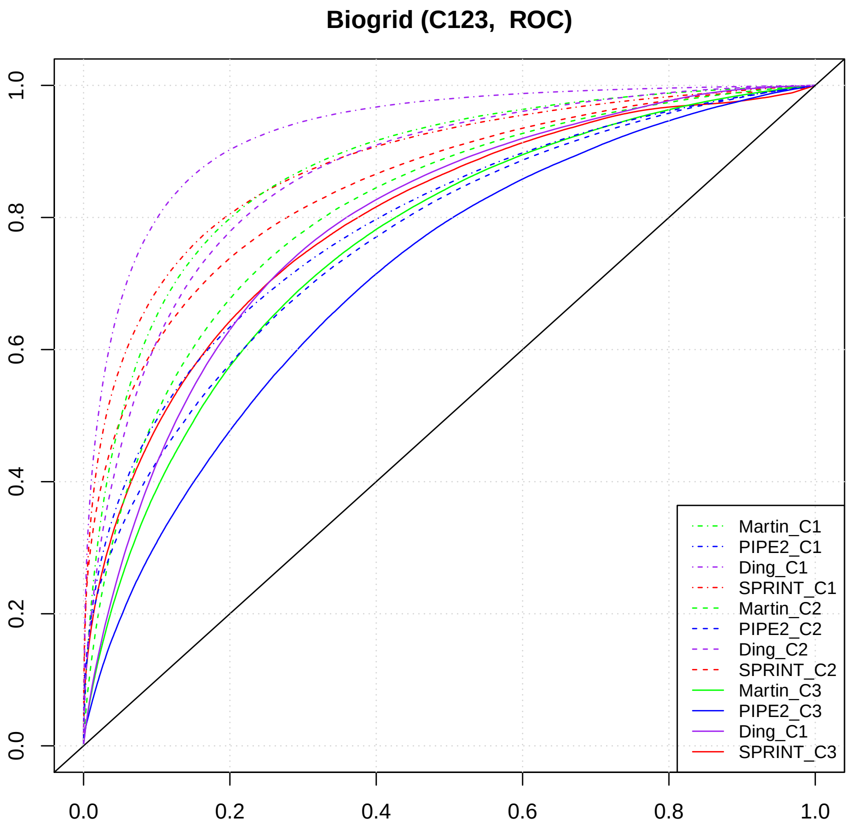
Biogrid (C123, ROC)
1.0
0.8
0.6
0.4
0.2
0.0
0.0
0.2
0.4
0.6
0.8
1.0
Martin_C1
PIPE2_C1
Ding_C1
SPRINT_C1
Martin_C2
PIPE2_C2
Ding_C2
SPRINT_C2
Martin_C3
PIPE2_C3
Ding_C1
SPRINT_C3

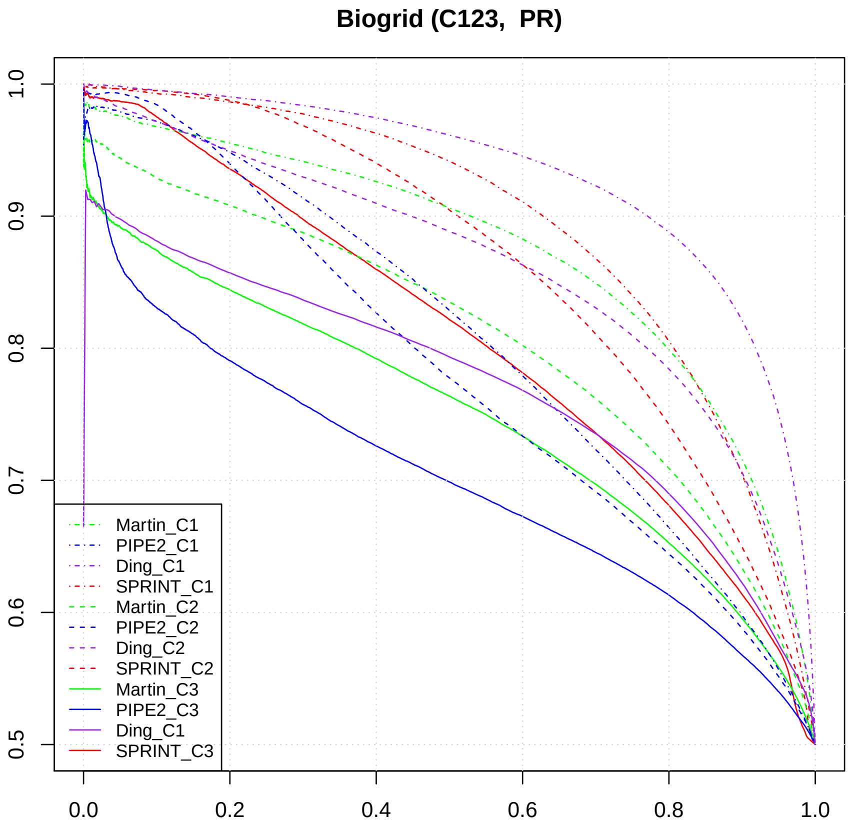
Biogrid (C123, PR)
1.0
0.9
0.8
0.7
0.6
0.5
0.0
0.2
0.4
0.6
0.8
1.0
Martin_C1
PIPE2_C1
Ding_C1
SPRINT_C1
Martin_C2
PIPE2_C2
Ding_C2
SPRINT_C2
Martin_C3
PIPE2_C3
Ding_C1
SPRINT_C3

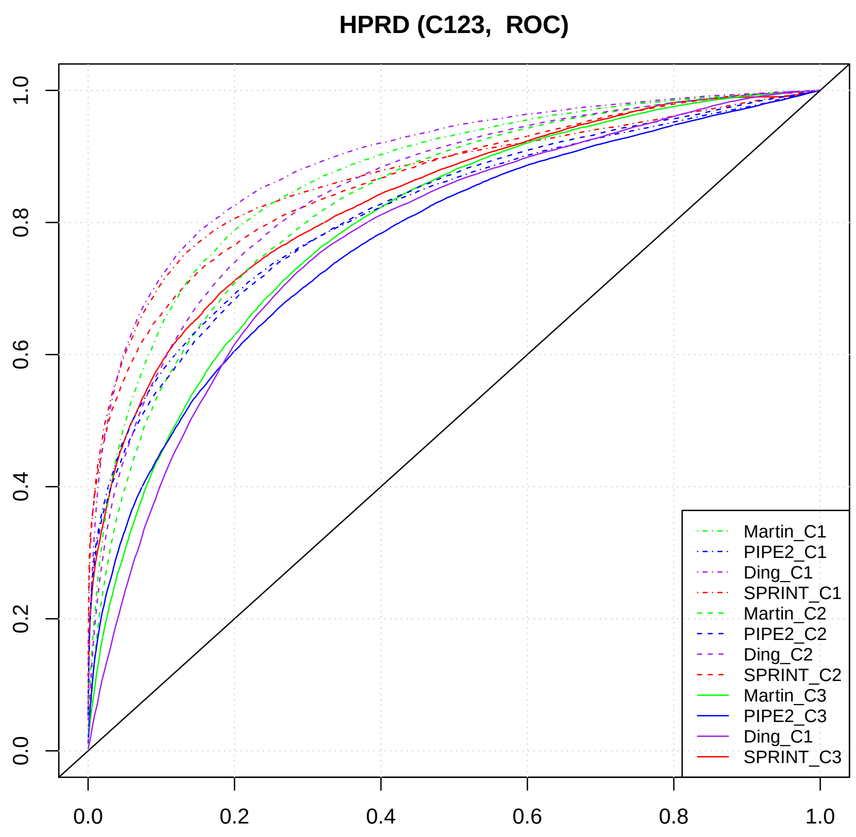
HPRD (C123, ROC)
1.0
0.8
0.6
0.4
0.2
0.0
0.0
0.2
0.4
0.6
0.8
1.0
Martin_C1
PIPE2_C1
Ding_C1
SPRINT_C1
Martin_C2
PIPE2_C2
Ding_C2
SPRINT_C2
Martin_C3
PIPE2_C3
Ding_C1
SPRINT_C3

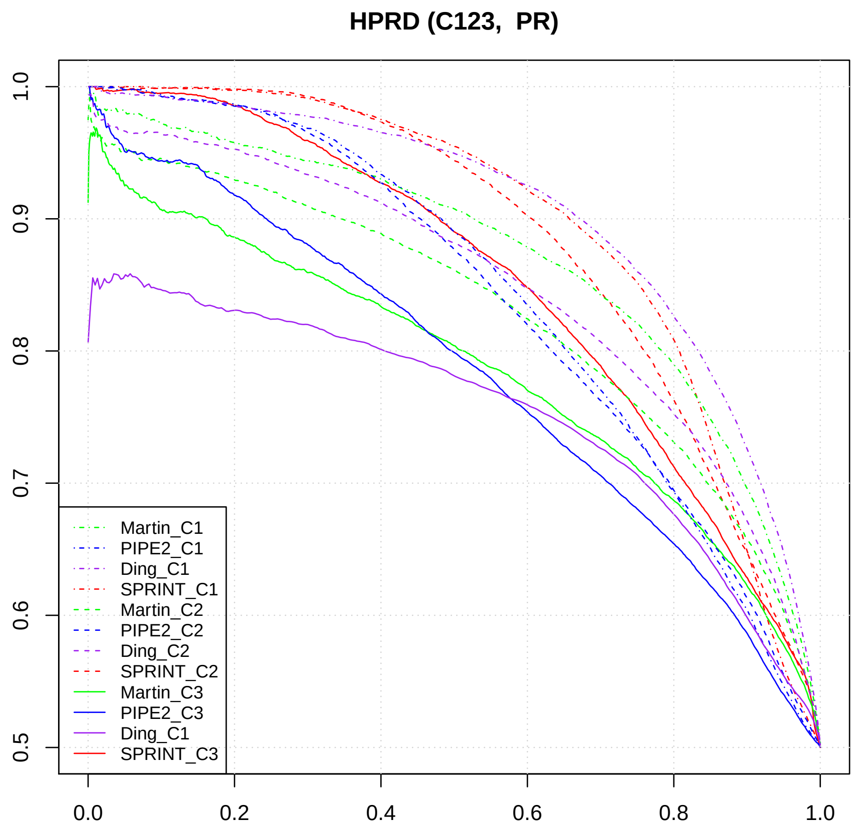
HPRD (C123, PR)
1.0
0.9
0.8
0.7
0.6
0.5
0.0
0.2
0.4
0.6
0.8
1.0
Martin_C1
PIPE2_C1
Ding_C1
SPRINT_C1
Martin_C2
PIPE2_C2
Ding_C2
SPRINT_C2
Martin_C3
PIPE2_C3
Ding_C1
SPRINT_C3

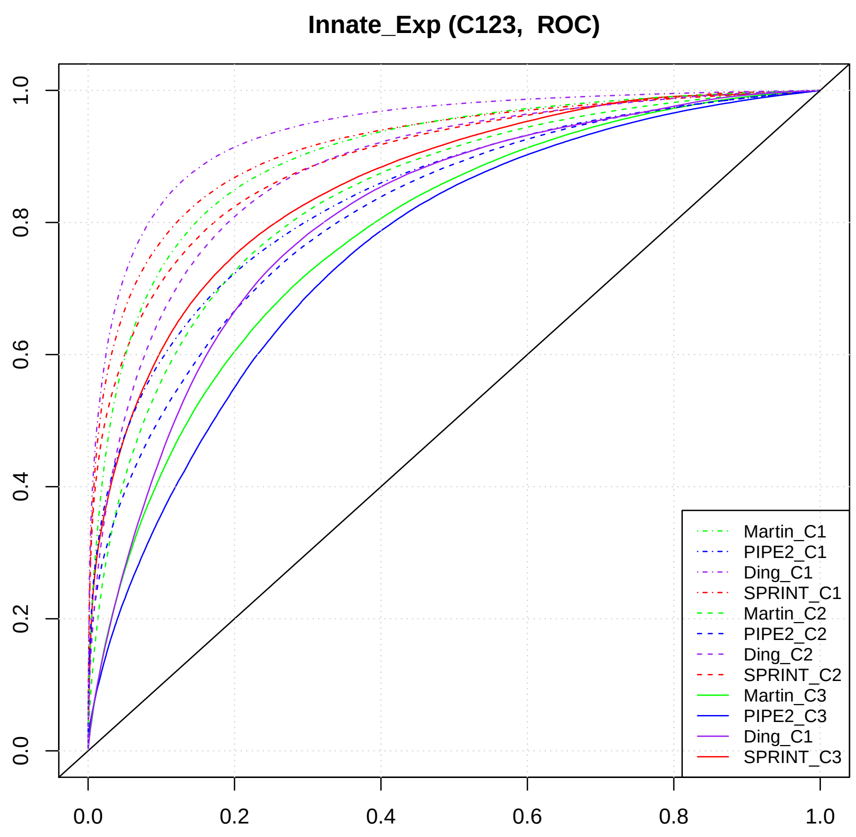
Innate_Exp (C123, ROC)
1.0
0.8
0.6
0.4
0.2
0.0
0.0
0.2
0.4
0.6
0.8
1.0
Martin_C1
PIPE2_C1
Ding_C1
SPRINT_C1
Martin_C2
PIPE2_C2
Ding_C2
SPRINT_C2
Martin_C3
PIPE2_C3
Ding_C1
SPRINT_C3

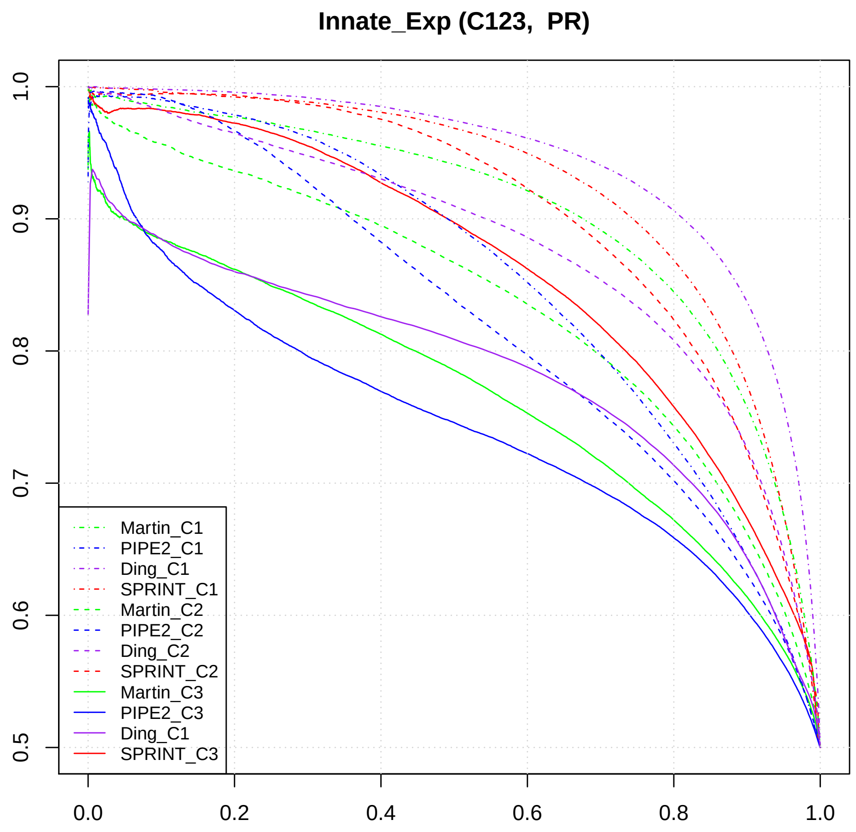
Innate_Exp (C123, PR)
1.0
0.9
0.8
0.7
0.6
0.5
0.0
0.2
0.4
0.6
0.8
1.0
Martin_C1
PIPE2_C1
Ding_C1
SPRINT_C1
Martin_C2
PIPE2_C2
Ding_C2
SPRINT_C2
Martin_C3
PIPE2_C3
Ding_C1
SPRINT_C3

Innate_Man (C123, ROC)

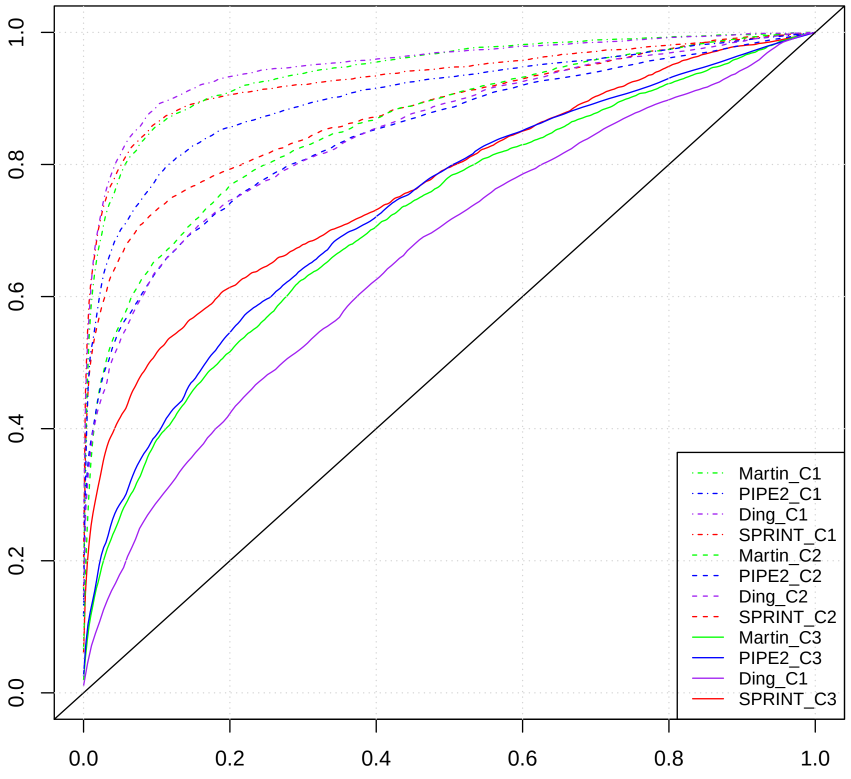

Innate_Man (C123, PR)

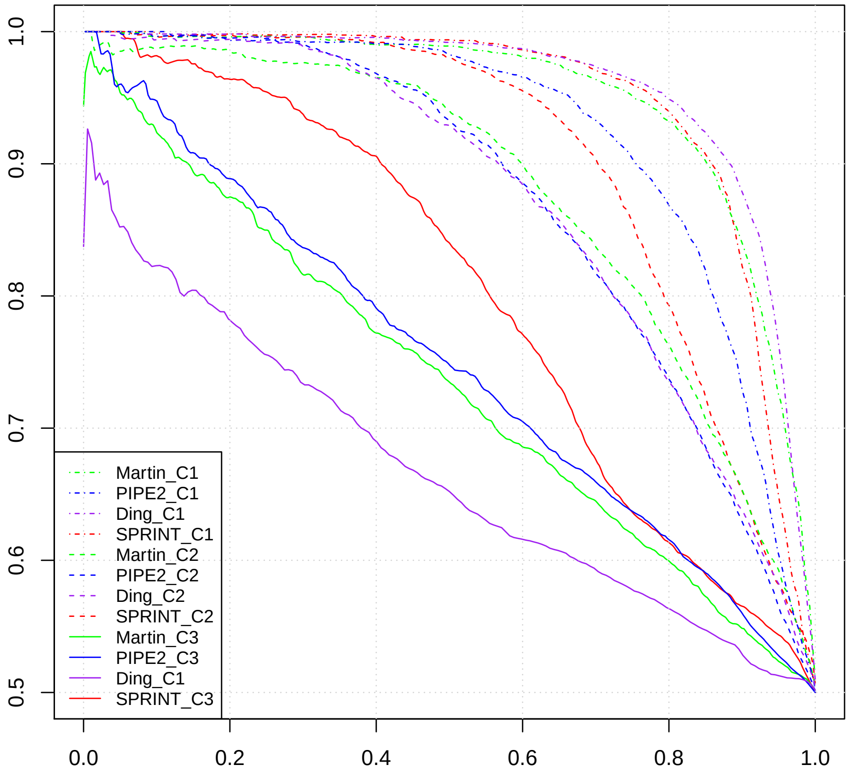

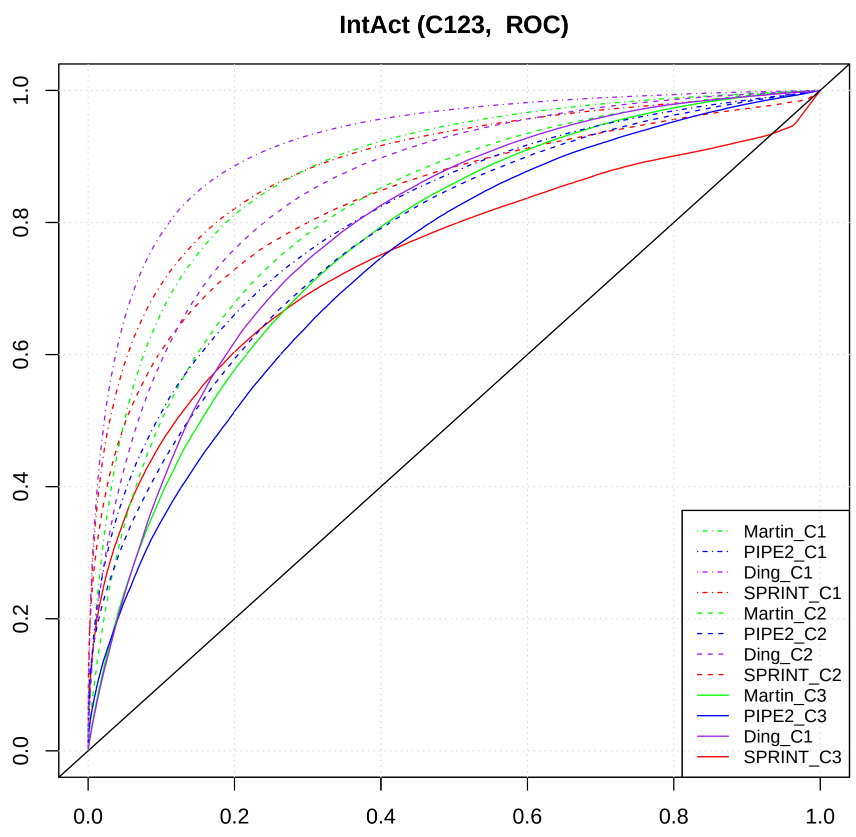
IntAct (C123, ROC)
1.0
0.8
0.6
0.4
0.2
0.0
0.0
0.2
0.4
0.6
0.8
1.0
Martin_C1
PIPE2_C1
Ding_C1
SPRINT_C1
Martin_C2
PIPE2_C2
Ding_C2
SPRINT_C2
Martin_C3
PIPE2_C3
Ding_C1
SPRINT_C3

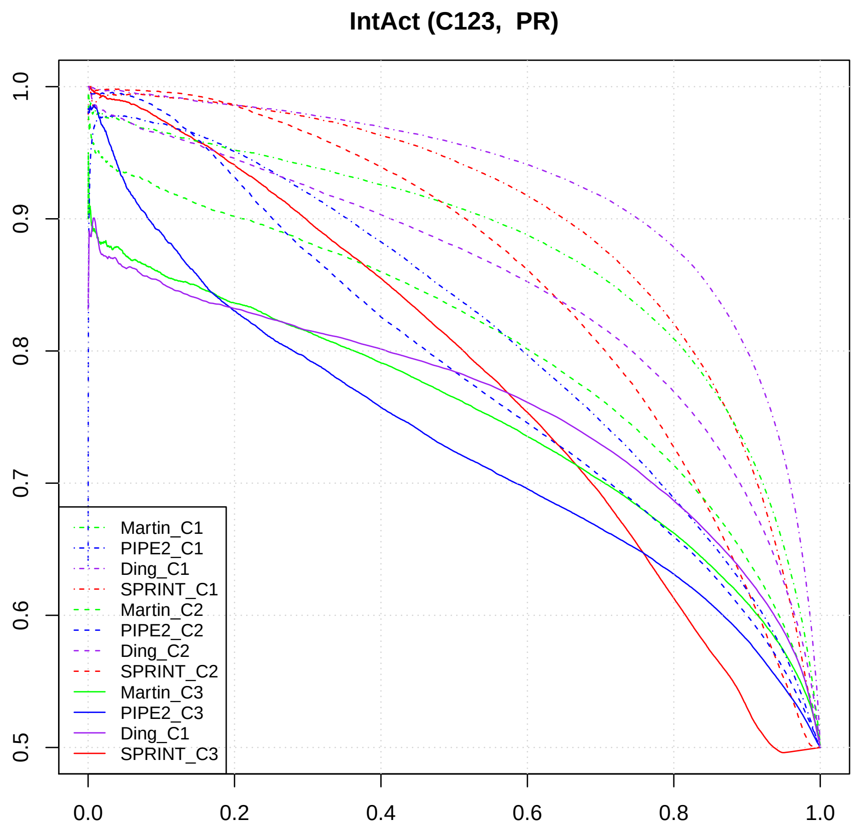
IntAct (C123, PR)
1.0
0.9
0.8
0.7
0.6
0.5
0.0
0.2
0.4
0.6
0.8
1.0
Martin_C1
PIPE2_C1
Ding_C1
SPRINT_C1
Martin_C2
PIPE2_C2
Ding_C2
SPRINT_C2
Martin_C3
PIPE2_C3
Ding_C1
SPRINT_C3

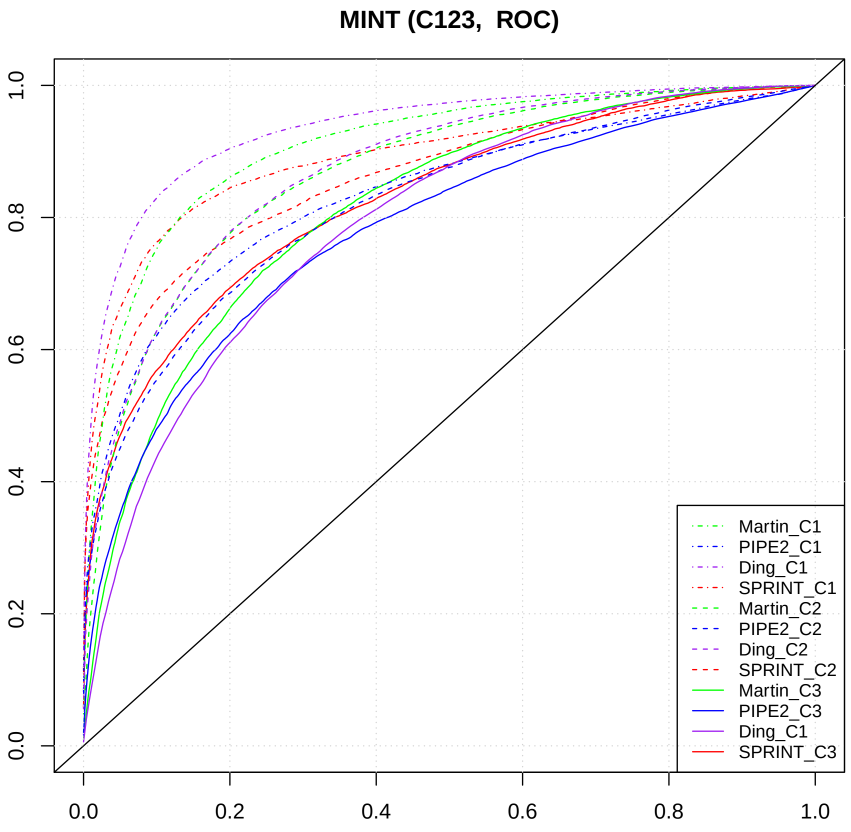
MINT (C123, ROC)
1.0
0.8
0.6
0.4
0.2
0.0
0.0
0.2
0.4
0.6
0.8
1.0
Martin_C1
PIPE2_C1
Ding_C1
SPRINT_C1
Martin_C2
PIPE2_C2
Ding_C2
SPRINT_C2
Martin_C3
PIPE2_C3
Ding_C1
SPRINT_C3

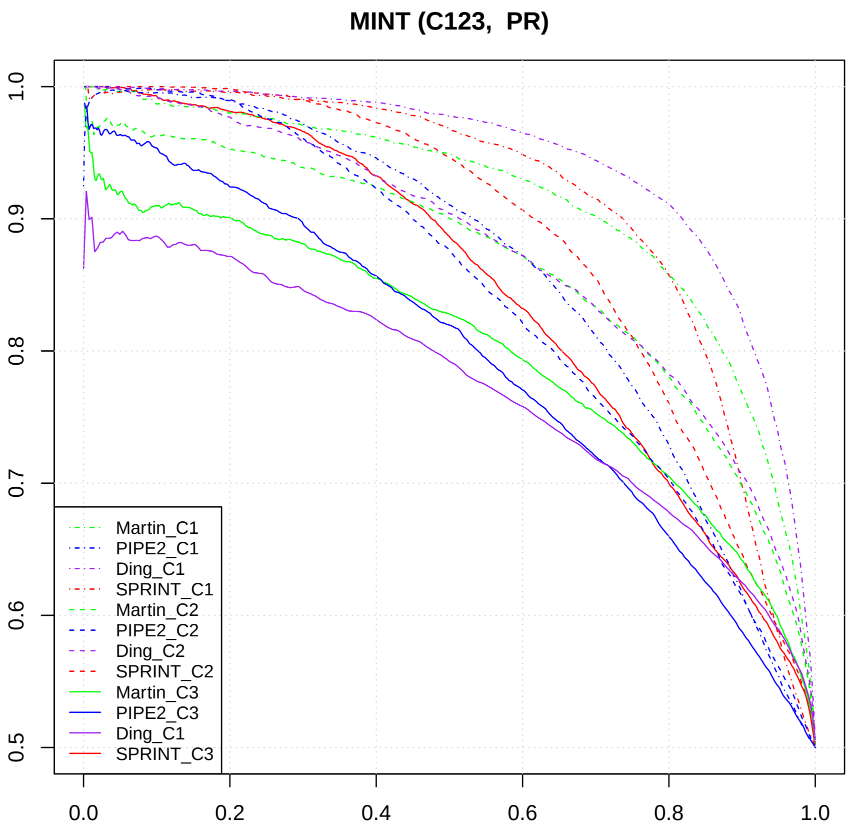
MINT (C123, PR)
1.0
0.9
0.8
0.7
0.6
0.5
0.0
0.2
0.4
0.6
0.8
1.0
Martin_C1
PIPE2_C1
Ding_C1
SPRINT_C1
Martin_C2
PIPE2_C2
Ding_C2
SPRINT_C2
Martin_C3
PIPE2_C3
Ding_C1
SPRINT_C3
